# Endoscopic transnasal transmaxillary approach to the upper parapharyngeal space and the skull base

**DOI:** 10.1007/s00405-019-05761-6

**Published:** 2019-12-16

**Authors:** Quan Liu, Huan Wang, Weidong Zhao, Xiaole Song, Xicai Sun, Hongmeng Yu, Dehui Wang, Juan C. Fernandez-Miranda, Carl H. Snyderman

**Affiliations:** 1 Department of Otolaryngology, Eye, Ear, Nose and Throat Hospital, Shanghai Medical College of Fudan University, 83 Fenyang Road, Shanghai, 200031 China; 2 Department of Neurosurgery, Stanford University Medical Center, Stanford, CA USA; 3 Department of Otolaryngology, University of Pittsburgh School of Medicine, Pittsburgh, PA USA

**Keywords:** Skull base, Endoscopy, Parapharyngeal space

## Abstract

**Purpose:**

Treatment of tumors arising in the upper parapharyngeal space (PPS) or the floor of the middle cranial fossa is challenging. This study aims to present anatomical landmarks for a combined endoscopic transnasal and anterior transmaxillary approach to the upper PPS and the floor of the middle cranial fossa and to further evaluate their clinical application.

**Methods:**

Dissection of the upper PPS using a combined endoscopic endonasal transpterygoid and anterior transmaxillary approach was performed in six cadaveric heads. Surgical landmarks associated with the approach were defined. The defined approach was applied in patients with tumors involving the upper PPS.

**Results:**

The medial pterygoid muscle, tensor veli palatini muscle and levator veli palatini muscle were key landmarks of the approach into the upper PPS. The lateral pterygoid plate, foramen ovale and mandibular nerve were important anatomical landmarks for exposing the parapharyngeal segment of the internal carotid artery through a combined endoscopic transnasal and anterior transmaxillary approach. The combined approach provided a better view of the upper PPS and middle skull base, allowing for effective bimanual techniques and bleeding control. Application of the anterior transmaxillary approach also provided a better view of the inferior limits of the upper PPS and facilitated control of the internal carotid artery.

**Conclusions:**

Improving the knowledge of the endoscopic anatomy of the upper PPS allowed us to achieve an optimal approach to tumors arising in the upper PPS. The combined endoscopic transnasal and anterior transmaxillary approach is a minimally invasive alternative approach to the upper PPS.

## Introduction

The parapharyngeal space (PPS) is described as an inverted pyramid with the base formed by the skull base and the apex pointing to the greater cornu of the hyoid bone; the PPS is limited by the pterygomandibular raphe anteriorly, by the inner fascia of the masticatory space and the deep lobe of the parotid gland laterally, and by the buccopharyngeal and alar fasciae medially. The posterior limit of the PPS is generally regarded to extend to the posterior fascia of the carotid sheath. The PPS is divided into pre- and poststyloid compartments by the tensor-vascular-styloid (TVS) fascia. Tumors arising from the PPS represent 0.5% of head and neck neoplasms [1]. The deep location of the PPS presents challenges in terms of diagnosis and management. The treatment of lesions arising from the upper PPS is challenging and usually requires an extended approach to avoid injury to adjacent neurovascular structures [1–3]. Traditionally, tumors arising from the PPS have been managed through a variety of lateral approaches, including transcervical, transoral, mandibular swing, transparotid, transmastoid, and infratemporal fossa (ITF) approaches and their combinations [4, 5].

Increasing literatures have presented the application of endoscopic endonasal surgery in managing lesions involving the upper PPS, with the advantage of reducing the incidence of functional and cosmetic morbidity related to open approaches [6, 7]. Van Rompaey et al. demonstrated that an endoscopic transmaxillary/transpterygoid approach provided a sufficient surgical window for tumor extirpation, obviating some of the morbidity associated with an open procedure; however, this approach was limited by its access to the petrous portion of the internal carotid artery (ICA) [2].

Sun and his colleagues compared the detailed anatomy of the PPS observed via transnasal transpterygoid and endoscopic-assisted transoral approaches [3]. However, the detailed endoscopic anatomy of the upper PPS from an endoscopic standpoint is still poorly understood; thus, the purpose of this study was to present the endoscopic anatomy of the upper PPS observed through a combined endoscopic transnasal and anterior transmaxillary approach and to further illustrate its clinical application.

## Materials and methods

All dissections were performed in the Surgical Neuroanatomy Laboratory at the University of Pittsburgh. This study was approved by the local institutional research committee. Six cadaveric specimens (12 sides) were used. Each cadaveric head was injected with colored silicone; red silicone for the arterial system and blue for the venous system. Endoscopes (0° and 30°) coupled with a high-definition camera and an AIDA HD system (Karl Storz GmbH and Co.) were used to provide excellent visualization [3]. Stepwise dissection via the combined endoscopic transnasal and anterior transmaxillary approach was performed to present the landmarks of the upper PPS and the middle skull base. The optimal approach obtained from the anatomical study was applied in patients with tumors arising from the upper PPS.

## Results

### Exposure of the sinonasal corridor

**Fig. 1:**
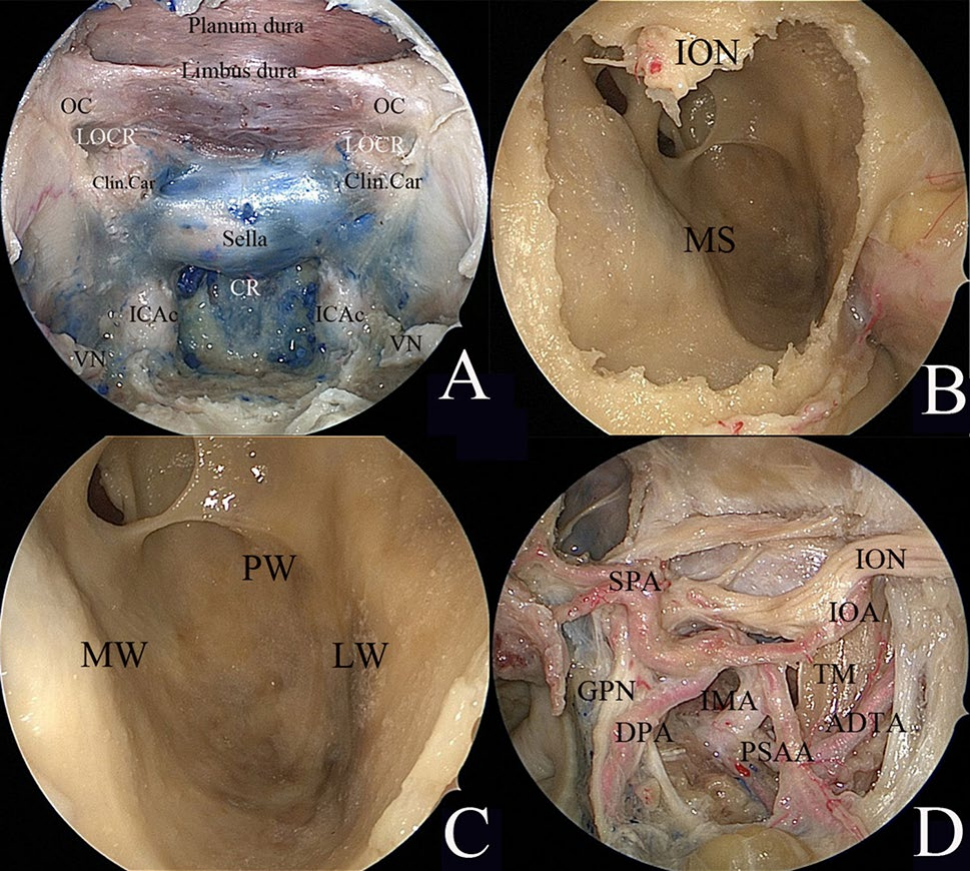
Endoscopic transnasal transmaxillary approach. **a** Endoscopic view of the midline skull base. **b** Removal of the anterior wall of the maxillary sinus. **c** Endoscopic transmaxillary approach to expose the maxillary sinus. **d** Endoscopic view of the anatomic structures in the pterygopalatine fossa and the infratemporal fossa. *ADTA* anterior deep temporal artery, *Clin. car.* clinoidal carotid artery, *CR* clival recess, *DPA* descending palatine artery, *GPN* greater palatine nerve, *ICAc* paraclival segment of the internal carotid artery, *IMA* internal maxillary artery, *IOA* inferior optic artery, *ION* inferior optic nerve, *LOCR* lateral opticocarotid recess, *LW* lateral wall, *MS* maxillary sinus, *MW* medial wall, *OC* optic canal, *PSAA* posterior superior alveolar artery, *PW* posterior wall, *SPA* sphenopalatine artery, *TM* temporal muscle, *VN* vidian nerve

Initially, uncinectomy and enlargement of the maxillary ostium were performed, followed by ethmoidectomy to expose the medial wall of the orbit. The ipsilateral middle turbinate was then removed, followed by transethmoidal sphenoidotomy. Landmarks of the paraclival carotid artery, optic nerve, pituitary, and cavernous sinus were identified to provide a panoramic view prior to embarking on the approach to the pterygopalatine fossa (PPF) and the ITF (Fig. 1a).

### Establishment of the endoscopic anterior transmaxillary corridor

To provide a complete view of the posterior wall of the maxillary sinus, the Caldwell-Luc procedure was performed. A horizontal incision 1.5 cm in length and 1 cm above the gingival mucosa was made on the ipsilateral anterior wall of the maxillary sinus, as described in our previous report [8]. Upper elevation of the soft tissue was limited to the infraorbital foramen, and the infraorbital nerve (ION) was identified (Fig. 1b).

The anterior wall was drilled to access the maxillary sinus. The course of the ION was observed from the junction of the roof, posterior wall, and lateral wall of the maxillary sinus along the roof (Fig. 1c).

### Exposure of the PPF and ITF

**Fig. 2:**
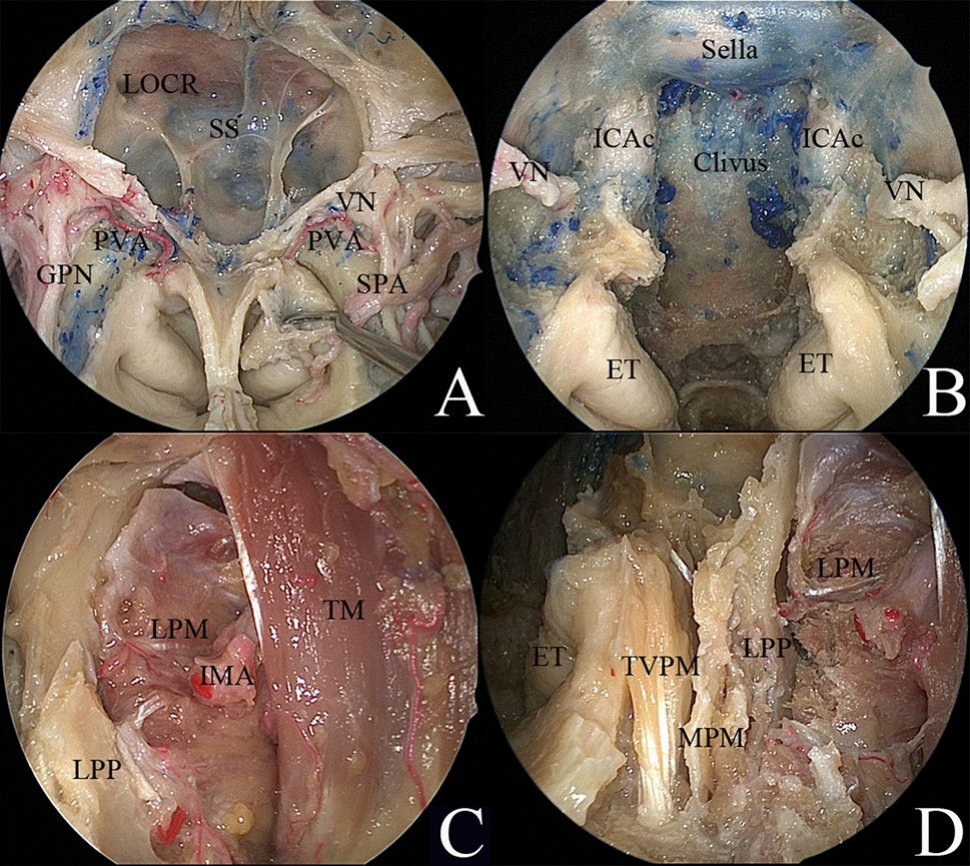
Endoscopic view of the transpterygoid approach. **a** Endoscopic view of the paramedian skull base. **b** Endoscopic view of the clivus. **c** The lateral pterygoid muscle and the temporal muscle were identified after removal of the vascular plexus in the infratemporal fossa. **d** The pterygoid base was removed by drilling to expose the pterygoid plates. The lateral and medial pterygoid muscles were detached from the lateral pterygoid plate. *ET* eustachian tube, *GPN* greater palatine nerve, *ICAc* paraclival segment of the internal carotid artery, *IMA* internal maxillary artery, *LOCR* lateral opticocarotid recess, *LPM* lateral pterygoid muscle, *LPP* lateral pterygoid plate, *MPM* medial pterygoid muscle, *PVA* palatovaginal artery, *SPA* sphenopalatine artery, *SS* sphenoid sinus, *TM* temporal muscle, *TVPM* tensor veli palatini muscle, *VN* vidian nerve

Endoscopic dissection of the PPF was performed starting by exposing the sphenopalatine artery (SPA) via removal of the crista ethmoidalis. The descending palatine artery and the greater palatine nerve were further exposed via removal of the perpendicular plate of the palatine bone. Kerrison rongeurs were used to remove the medial posterior wall of the maxillary sinus. During this stage, the endoscopic anterior transmaxillary approach was used to expose the lateral part of the ITF. Upon removal of the periosteum, the fat from the PPF was visible. Careful removal of the fat revealed a complex vascular network. The internal maxillary artery (IMA) and its branches were identified (Fig. 1d). The soft tissues of the PPF were displaced laterally. The palatovaginal artery and vidian canal nerve were identified and cut (Fig. 2a, b). The lateral pterygoid muscle (LPM) and the temporalis muscle (TM) were identified after the removal of the vascular plexus (Fig. 2c). The pterygoid base was drilled to expose the pterygoid plates. The lateral and medial pterygoid muscles were detached from the lateral pterygoid plate (Fig. 2d).

### Exposure of the upper PPS and the middle cranial base

**Fig. 3:**
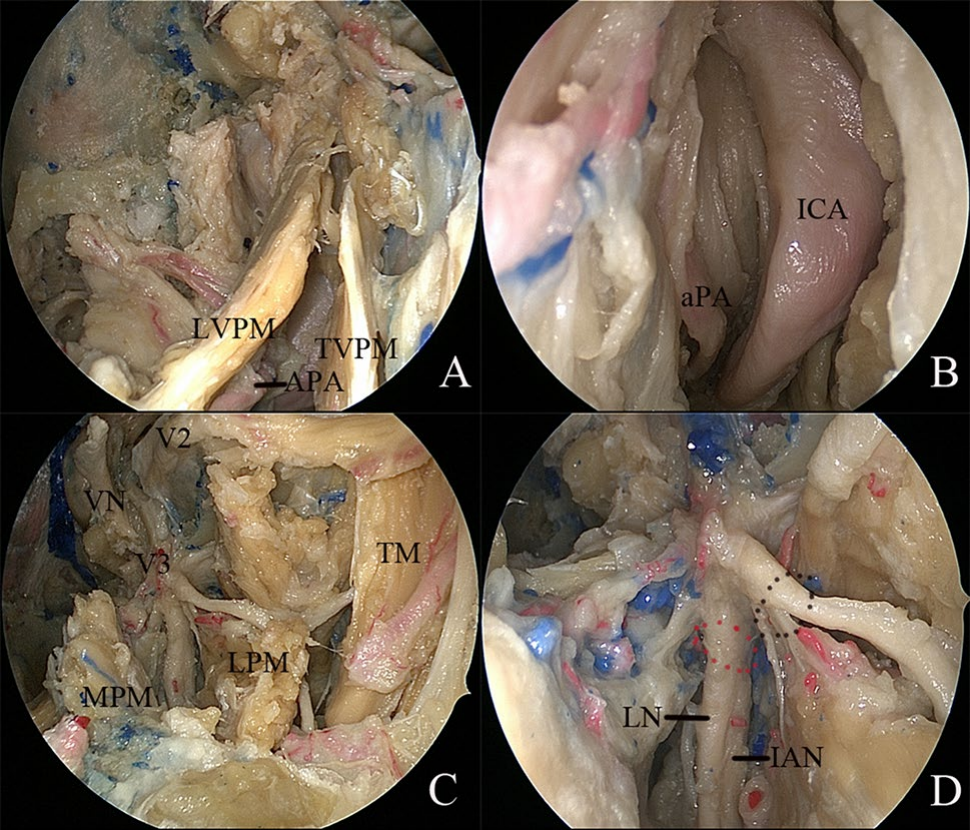
Endoscopic transnasal transmaxillary approach to the upper parapharyngeal space and middle skull base. **a** The medial space of the upper parapharyngeal space was exposed. **b** The ascending palatine artery and internal carotid artery became visible in the poststyloid compartment of the upper parapharyngeal space. **c** The lateral space of the upper parapharyngeal space was exposed. **d** Branches of the trigeminal nerve were demonstrated. *APA* ascending palatine artery, *aPA* ascending pharyngeal artery, *IAN* inferior alveolar nerve, *ICA* internal carotid artery, *LN* lingual nerve, *LPM* lateral pterygoid muscle, *LVPM* levator veli palatini muscle, *MPM* medial pterygoid muscle, *TM* temporal muscle, *TVPM* tensor veli palatini muscle, *VN* vidian nerve, *V2* maxillary nerve, *V3* mandibular nerve, black dotted circle, the anterior root of the mandibular nerve; red dotted circle, the posterior root of the mandibular nerve

The pterygoid process inferior to the level of the nasal floor was drilled. The fat pad of the upper PPS was encountered between the tensor veli palatini muscle (TVPM) and medial pterygoid muscle (MPM), which formed the lateral space of the upper PPS [9]. Retracting the MPM and TVPM laterally was helpful to expose the levator veli palatini muscle (LVPM). The soft tissue between the TVPM and superior pharyngeal constrictor muscle was removed to expose the medial space of upper PPS, as described in our previous report [3]. The medial space of the upper PPS was dissected posteriorly to access the poststyloid compartment. During this part of the dissection, the branches of the ascending palatine artery became visible (Fig. 3a). Posterior dissection of the carotid sheath made it possible to expose the parapharyngeal segment of the ICA and ascending pharyngeal artery (Fig. 3b). Detachment of the LPM from the lateral pterygoid plate and the greater wing of the sphenoid was performed to expose the foramen ovale (FO). V3 and its corresponding branches were identified (Fig. 3c, d).

Therefore, maximal exposure achieved using the combined endoscopic transnasal and anterior transmaxillary approach to the upper PPS and the floor of the middle cranial fossa included exposure of the temporomandibular joint laterally, the parapharyngeal ICA posteriorly, the greater wing of the sphenoid superiorly and the floor of the maxillary sinus inferiorly.

### Illustrative cases

#### Case 1: recurrent nasopharyngeal carcinoma with upper PPS involvement

**Fig. 4:**
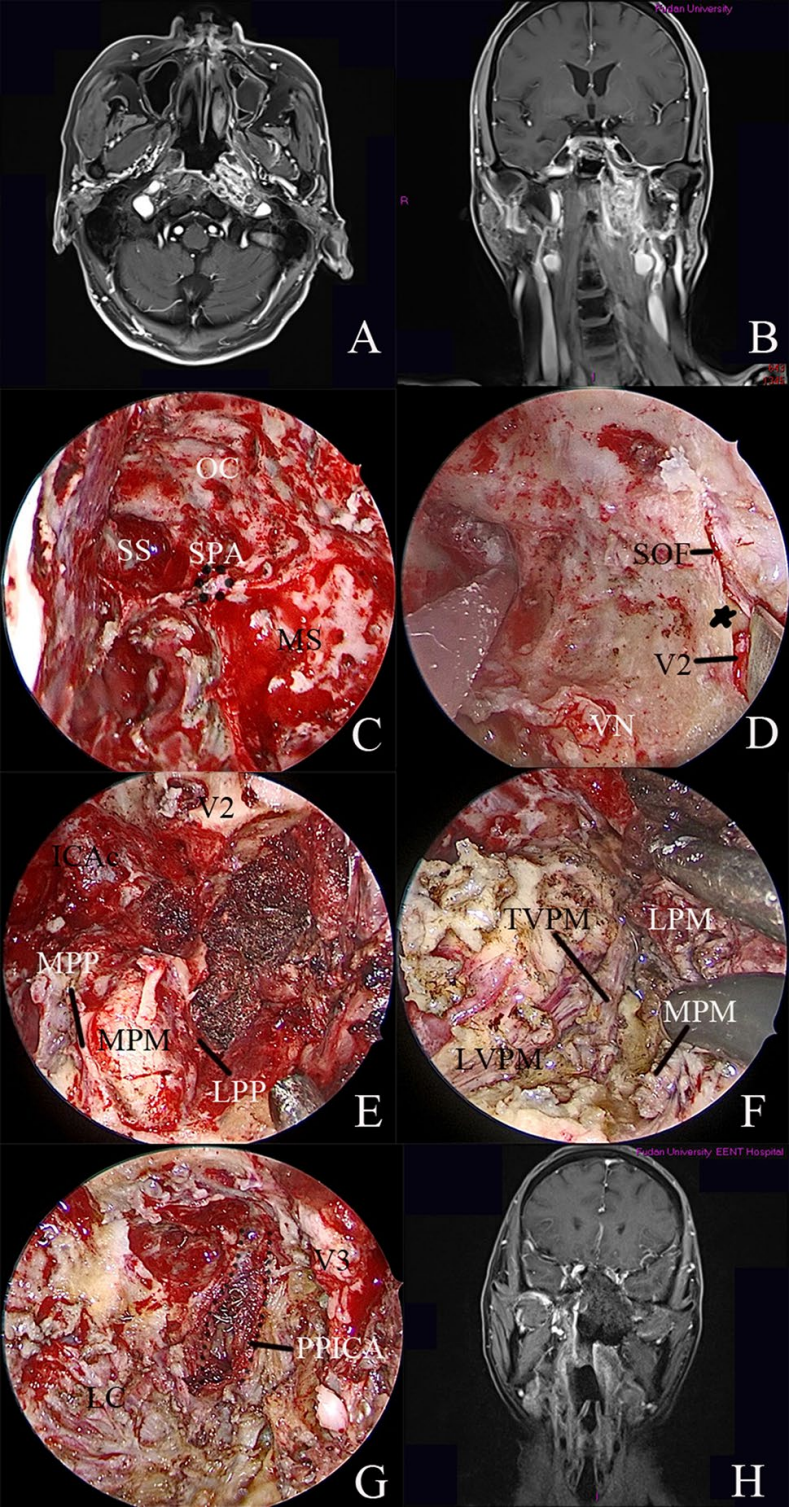
*Case 1* Recurrent nasopharyngeal carcinoma. **a**, **b** MRI with gadolinium revealed that the mass was located in the left nasopharyngeal fossa, extending into the upper parapharyngeal space with involvement of the posterior wall of the nasopharynx, longus capitis muscle and parapharyngeal and petrous internal carotid arteries. **b** The internal carotid artery was occluded after the BOT. **c** Anteroposterior ethmoidectomy, maxillary antrostomy, and sphenoidotomy were completed to expose the guiding landmarks for providing a panoramic view prior to embarking on the approach to the pterygopalatine fossa and the infratemporal fossa. **d** Endoscopic view of the middle skull base. **e**, **f** The upper parapharyngeal space was dissected via the endoscopic transnasal transmaxillary approach. **g** The intrapetrous and parapharyngeal internal carotid arteries were exposed. **h** Postoperative MRI indicated complete resection of the recurrent nasopharyngeal carcinoma. *OC* optic canal, *ICAc* paraclival segment of the internal carotid artery, *LC* longus capitis, *LPM* lateral pterygoid muscle, *LPP* lateral pterygoid plate, *LVPM* levator veli palatini muscle, *MPM* medial pterygoid muscle, *MPP* medial pterygoid plate, *PPICA* parapharyngeal internal carotid artery, *SPA* sphenopalatine artery, *SS* sphenoid sinus, *SOF* superior orbital fissure, *TVPM* tensor veli palatini muscle, *VN* vidian nerve, *V2* maxillary nerve, *V3* mandibular nerve, black dotted line, the parapharyngeal internal carotid artery, asterisk: maxillary strut

A 59-year-old man with recurrent nasopharyngeal carcinoma presented to Eye, Ear, Nose and Throat Hospital, Shanghai Medical College of Fudan University in November 2018, reporting bloody rhinorrhea for 2 months. Magnetic resonance imaging (MRI) with gadolinium revealed that the mass was located in the left nasopharyngeal fossa, extending into the upper PPS with the involvement of the posterior wall of the nasopharynx, longus capitis muscle and parapharyngeal and petrous ICA (Fig. 4a). To achieve radical extirpation, the involved ICA was occluded after the balloon occlusion test (Fig. 4b). The combined endoscopic transnasal and anterior transmaxillary approach were performed on the patient. Initially, endoscopic resection of the tumor in the nasal cavity was performed, and then anteroposterior ethmoidectomy, maxillary antrostomy, and sphenoidotomy were completed to expose the guiding landmarks for the panoramic view prior to embarking on the approach to the PPF and ITF (Fig. 4c). The anterior wall of the ipsilateral maxillary sinus was drilled with preservation of the ION, allowing for bimanual techniques. The posterior wall of the maxillary sinus was drilled to expose the PPF and ITF. After identification and cauterization of the SPA, the PPF was retracted laterally to expose the vidian nerve. The FO and maxillary nerve were dissected. The maxillary strut was revealed between the superior orbital fissure and the maxillary nerve (Fig. 4d). Anteroposterior drilling along the vidian nerve was performed to expose the anterior genu of the ICA. The pterygoid process was drilled inferiorly to the level of the nasal floor. The lateral and medial pterygoid plates were then exposed, followed by removal of the LPM and MPM (Fig. 4e). The TVPM and LVPM served as the guiding landmarks (Fig. 4f). As described by Shen in 2016, the parapharyngeal ICA (PPICA) was located in the same sagittal plane as the TVP [10]. Dissection was continued posteriorly along the fascial plane of the TVP until the PPICA was visualized. The inferior surface of the petrous bone was removed by drilling to expose the intrapetrous ICA (Fig. 4g).

Postoperative MRI revealed total resection of the tumor (Fig. 4h), and the patient recovered well after the surgery. The patient underwent regular follow-up examinations.

#### Case 2: recurrent sinonasal adenoid cystic carcinoma with the involvement of the ITF, the upper PPS and the cavernous sinus

**Fig. 5:**
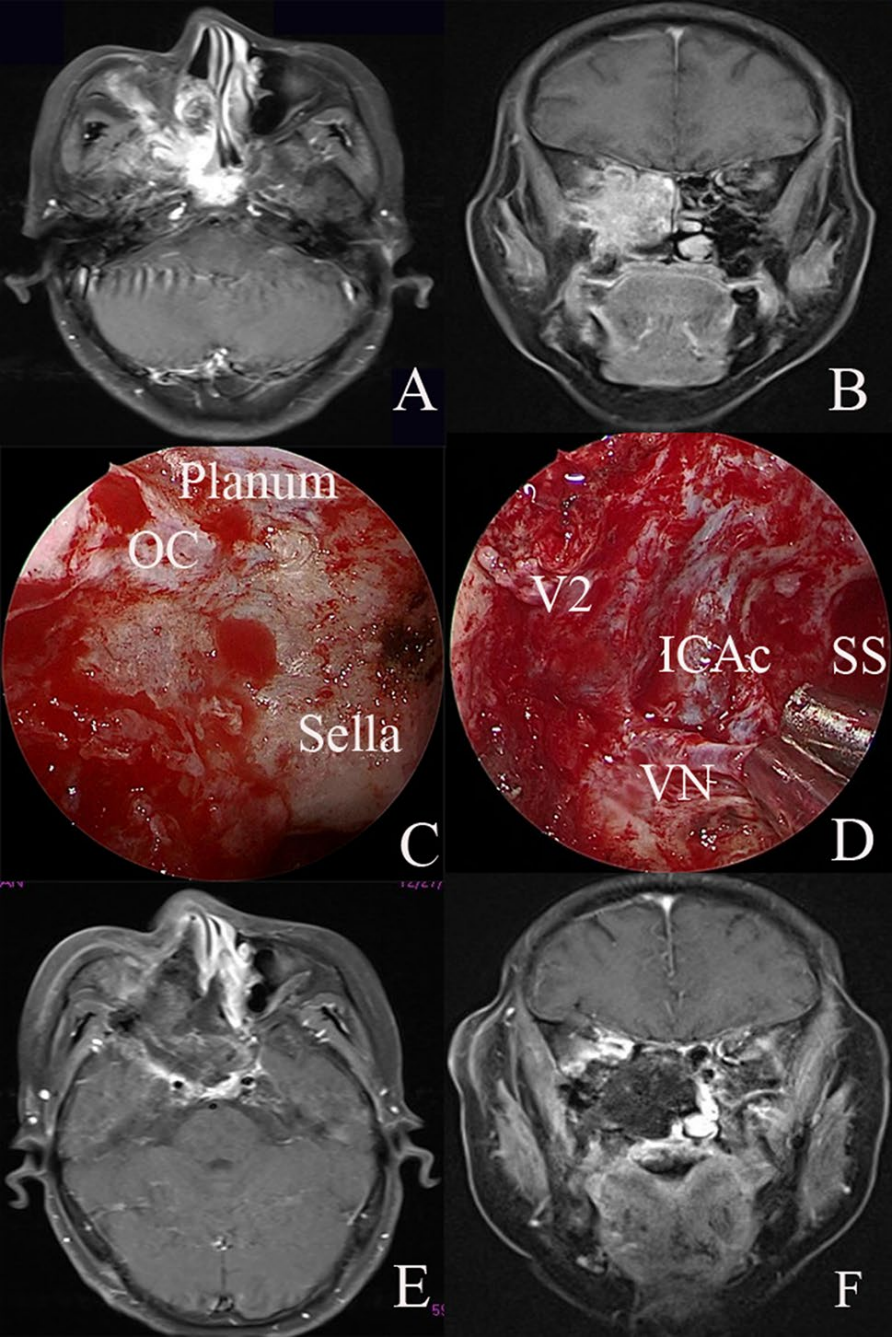
*Case 2* Recurrent sinonasal adenoid cystic carcinoma. **a**, **b** Preoperative MRI indicated that the tumor involved the infratemporal fossa, upper parapharyngeal space, cavernous sinus and middle skull base. **c** The sphenoid sinus and lateral wall structures, including the paraclival internal carotid artery and optic nerve, were exposed. **d** The paraclival internal carotid artery was located. The foramen ovale was located and showed tumor involvement. The maxillary nerve was resected by drilling off the bone, and the floor of the middle cranial fossa was exposed. **e**, **f** Postoperative MRI demonstrated complete resection of the tumor. *OC* optic canal, *ICAc* paraclival segment of the internal carotid artery, *VN* vidian nerve, *V2* maxillary nerve, *SS* sphenoid sinus

A patient with recurrent sinonasal adenoid cystic carcinoma was referred to Eye, Ear, Nose and Throat Hospital, Shanghai Medical College of Fudan University in December 2017. MRI revealed that the tumor involved the ITF, the upper PPS and the cavernous sinus (Fig. 5a, b). The combined endoscopic transnasal and anterior transmaxillary approach were performed on the patient. The tumor in the sinus was first resected via the endoscopic transnasal approach. The sphenoid sinus, lateral wall structures, including paraclival ICA and optic nerve, were exposed (Fig. 5c). The vidian nerve was identified and drilled posteriorly to reveal the paraclival ICA. The FO was located and showed tumor involvement. The maxillary nerve was resected by drilling off the bone, and the floor of the middle cranial fossa was exposed (Fig. 5d). The lingual process was drilled between the paraclival ICA and V3. After complete removal of the tumor, the surgical field was covered using a contralateral vascularized nasal septal flap.

Postoperative MRI revealed complete tumor removal (Fig. 5e, f), and the patient recovered well after the surgery. The patient underwent regular follow-up examinations.

## Discussion

The PPS is an inverted pyramidal-shaped potential space with the base formed by the skull base and the apex pointing to the greater cornu of the hyoid bone. The fascia running posteriorly from the styloid process to the TVPM divides the PPS into the prestyloid (anterior) and poststyloid (posterior) compartments [1, 5]. Tumors in the prestyloid space are most commonly of a deep parotid gland origin, while tumors in the poststyloid space usually arise from neurovascular structures, including the carotid artery, internal jugular vein, cranial nerves IX–XII and sympathetic chain [11–13].

Multiple approaches have been reported for accessing tumors located in the superior aspect of the PPS. Transcervical approaches have traditionally been used for the management of lesions in the PPS. Among these, the subtemporal preauricular infratemporal approach has been commonly practiced and provides good access to PPS tumors with good exposure of blood vessels and nerves, especially the exposure of the petrous portion of the ICA. However, it requires a larger surgical window, which causes greater trauma [2, 14, 15].

Recently, with improving endoscopic techniques, transnasal endoscopic approaches have been preferred by surgeons, reducing the functional and cosmetic morbidity related to traditional open approaches. Varieties of endoscopic transnasal approaches to the PPS have been reported, including endoscopic transnasal transmaxillary/transpterygoid approaches, which provide improved access to the anterior and medial portions of the superior PPS [2]. The endoscopic prelacrimal approach is also commonly utilized to access the lateral ITF and the floor of the middle cranial fossa [16, 17]. However, the above-mentioned approaches are limited by the freedom of instrument movement [18, 19]. As demonstrated in our study, the combined endoscopic transnasal and anterior transmaxillary approach provides direct access to the upper PPS. Furthermore, this combined approach facilitates bimanual techniques as a result of the larger freedom of instrument movement, which is critical in endoscopic transnasal skull base surgery for the effective control of catastrophic bleeding.

The combined endoscopic transnasal and anterior transmaxillary approach to the upper PPS can be achieved without skull base resection or brain retraction. The approach is applicable to tumors located in the upper PPS, which is difficult to access using the traditional transcervical approach. An anatomical study of the PPS using an endoscopic transnasal transmaxillary transpterygoid approach was reported in 2010 by Taniguchi and Kohmura, who indicated that the approach would be restricted to benign tumors and inflammatory processes or limited-sized malignant tumors [9]. In this approach, the posterior portion of the nasal septum is sacrificed to allow bimanual techniques, which, is not sufficient for accessing the far lateral ITF and floor of the middle cranial fossa from the contralateral side. The narrow and deep corridor involved in the endoscopic transnasal transmaxillary transpterygoid approach limits the exposure of the carotid artery and jugular vein in the upper portion of the PPS. Furthermore, the purely transnasal transpterygoid approach limits instrument movement in the proximal surgical field. The combined endoscopic transnasal and anterior transmaxillary approach described in our manuscript are very helpful for exposing a large surgical field in the upper portion of the PPS and the middle skull base.

We must keep in mind that the approach we report is confined to managing tumors that are located mainly in the upper portion of the PPS. If the tumor involves the lower lateral portion of the PPS, a conventional transcervical or transoral approach should be applied to gain access to the PPS [20, 21].

When the endoscopic transnasal approach is used to treat the lesions in the upper PPS, the extra complications of nasal cavity, including nasal bleeding, adhesion, nasal dryness, and crusting, may occur. However, it should be remembered that the endoscopic transnasal approach is not less radical approach and difficulty in controlling hemorrhage, especially from the ICA. A major complication of the endoscopic transnasal approach to the upper PPS is intraoperative ICA injury. Thus, identification and protection of the ICA are crucial during the procedure. The important guiding landmarks include the vidian nerve and the eustachian tube. The vidian nerve, pointing to the anterior genu of the ICA, was first exposed by dissecting the PPF. Once the vidian nerve was identified, the superolateral foramen rotundum was located, which further guided exposure of the middle skull base. The eustachian tube served as a reliable anatomic landmark indicating the location of the carotid canal after pterygoid process resection, as described in previous reports [22, 23].

Traditionally, lateral and anterior approaches have been reported to treat the lesions in the upper PPS, which provide a wide window of exposure to address the surgical target but entail significant morbidity, including hearing loss, dysfunction of facial nerve, and dental malocclusion. Anterior approaches can carry an increased risk of facial deformity together with infra-orbital nerve and lacrimal dysfunctions [24]. In the past few years, improvement of endoscopic instruments and neuronavigation systems, the endoscopic endonasal transpterygoid transmaxillary approach provides a new feasible corridor for the treatment of tumors affecting the upper PPS. Compared to open approaches, endoscopic endonasal approach not just reduces functional and cosmetic morbidity but also provides a well illuminated, magnified, and multiangled view of the surgical field. The major drawbacks of endoscopic surgery in the upper PPS are the difficulty in controlling hemorrhage from the ICA, and the difficulty in physically accessing the lesion as the dissection proceeds more and more laterally [25].

## Conclusions

The optimal approach to the upper PPS should be guided by features of the lesion, including the tumor location, tumor size and extent of ICA involvement. It should be noted that the surgeon’s experience and preferences are also critical factors influencing the selection of an approach. The approach we present here is an alternative and effective method for accessing the upper PPS. The approach offers sufficiently wide exposure to ensure complete removal using bimanual techniques and facilitates the control of bleeding, especially bleeding from the ICA.
